# Exploring the Relationship Between Non-institutionalized Political Participation and Political Efficacy Among High School Students

**DOI:** 10.1007/s10964-025-02186-9

**Published:** 2025-04-21

**Authors:** Pascal Alscher, Costa Jana

**Affiliations:** 1 Center for Research on Education and School Development (IFS), TU Dortmund University, Dortmund, Germany; 2 Leibniz Institute for Educational Trajectories, Bamberg, Germany

**Keywords:** Adolescence, Civic engagement, Cross-lagged panel model, Political efficacy, Political participation

## Abstract

Understanding the interplay between political participation and political efficacy is crucial for fostering democratic engagement. This two-wave panel study investigated the potentially reciprocal relationship between non-institutionalized political participation and internal and external political efficacy among adolescents. The study drew on a German sample of 444 students from Grade 7 and Grade 8 (wave 1: *M*_age_ = 12.94, *SD*_age_ = 0.61; 46.9% female). It examined whether and how internal and external political efficacy are related to participation and whether participation, in turn, is related to political efficacy over time. Results revealed low stability for external political efficacy and non-institutionalized participation and moderate stability for internal political efficacy between Grade 7 and Grade 8. When students’ demographics were controlled for, internal political efficacy in Grade 7 was positively related to non-institutionalized participation in Grade 8, but no significant effects of participation on efficacy were observed. This study provides initial insights into the relationship between open democratic practices, like petitioning, protesting, and boycotting, and adolescents’ perceptions of political efficacy. Results suggested that the transformative potential of participatory activities may depend on specific conditions. The findings highlight the need for further research to explore these dynamics longitudinally and across different forms of participation.

## Introduction

Early engagement in diverse political activities may boost youth confidence in their political abilities but can also undermine efficacy if they perceive political systems as unresponsive. Understanding these dynamics seems crucial for comprehending the complex interplay between youth political participation and their perceptions of political agency. While much research explores what drives participation (Levy & Akiva, 2019), its consequences remain understudied, particularly its role as a democratic learning opportunity (Zvulun & Harel, 2018). Early political experiences have the potential to influence long-term engagement (Neundorf & Smets, 2017), yet access to such opportunities is unequal, reinforcing disparities in political socialization (Hoskins & Janmaat, 2016). Examining how participation influences political efficacy can inform strategies to strengthen democratic engagement. Though efficacy is often seen as a driver of participation (Chan & Mak, 2020), evidence suggests participation can also enhance efficacy (Šerek et al., 2017), particularly through non-institutionalized actions like petitioning, protesting, and boycotting, which provide meaningful agency-building experiences (Pickard, 2022). This study examines the bidirectional relationship between non-institutionalized political participation and political efficacy in adolescence, offering insights into the developmental foundations of democratic engagement.

### Political Participation and Political Efficacy

Political participation is fundamental to the functioning of democratic societies, enabling citizens to influence political processes. It includes various activities through which individuals actively engage in political life. Generally, political participation can take place in institutionalized and non-institutionalized forms. Unlike institutionalized participation, which is embedded in formal organizations like political parties or government institutions, non-institutionalized participation, such as petitions, demonstrations, and boycotts, are often driven by grassroots initiatives. These activities reflect a broader shift towards decentralized and self-directed engagement, where individuals seek to influence political and social issues outside formal institutions (Slavina, 2020). For young people, who often face barriers to accessing traditional forms of political participation, such as voting due to age restrictions, these other activities could offer a valuable alternative for political participation (Stockemer & Sundström, 2024). For example, demonstrations and social movements, such as climate marches or anti-corruption protests, mobilize large groups to visibly and vocally demand change (Schürmann, 2024). Petitions, whether online or offline, allow individuals to collectively call for specific actions or reforms, often drawing attention from policymakers and the public. Boycotts leverage consumer power, pressuring companies or governments to change their practices by refusing to purchase goods or services. These forms of non-institutionalized participation enable individuals and communities to engage in the political process on their own terms, creating a dynamic and responsive form of democracy (Micheletti & Stolle, 2012). While political participation reflects individuals’ engagement in political processes, an equally important aspect of democratic life is political efficacy.

Political efficacy is a vital aspect of citizenship in that it reflects the confidence citizens have of their sway over a political system (Kahne & Westheimer, 2006). It is generally recognized that political efficacy consists of two main components: internal and external efficacy (e.g., Alscher et al., 2023). Internal political efficacy involves an individual’s belief in his or her capacity to understand and influence political processes; this concept relates closely to “self-efficacy.” It includes the confidence to grasp political facts and processes and one’s belief that they can indeed shape political outcomes (Reichert, 2016). External political efficacy entails the belief that government officials care about and respond to what citizens say. This belief is strengthened by the idea that there is power in numbers, making collective actions more noticeable and likely to be addressed by governmental entities. Thus, individuals who belong to a strong, cohesive community are more likely to believe that their collective concerns will be heard by the government, particularly when they present a unified message (Anderson, 2010).

Both political efficacy and participation are shaped by an individual’s background. Various factors, such as gender, immigration background, and school track, influence how individuals engage with political life and develop political efficacy. Immigration background is a relevant factor because political socialization often occurs within families, and children of immigrant parents may have different levels of exposure to political knowledge and civic norms. This is evident in German federal elections, where, after controlling for voter eligibility, the participation gap was around 15 percent points in favor of people without an immigration background (Spies et al., 2020). Similarly, gender differences in political efficacy and participation are well-documented. Studies show that young men and women do not always engage in political activities to the same extent. Young men participate more often in institutionalized forms of political participation, whereas young women are more active in petitioning, boycotting, and volunteering (Grasso & Smith, 2022). Moreover, gendered socialization processes and structural barriers can influence the extent to which individuals perceive themselves as politically efficacious (Wolak, 2020). Finally, school track plays a crucial role in shaping political knowledge and engagement. The distinction between educational tracks is particularly relevant in the context of a German study, such as this, because the German secondary education system is divided into vocationally oriented schools (*Hauptschule* and *Realschule*) and academic-track schools (*Gymnasium*). In most German federal states, the transition from elementary to secondary education occurs at around age 10, with students being assigned to distinct educational tracks based on their academic performance, teacher recommendations, and parental input. Research from Germany has indicated that students attending academic-track schools tend to have higher levels of political knowledge, a stronger sense of political efficacy, and greater trust in political institutions compared to students in non-academic tracks (Wallrich et al., 2021). These differences may be attributable to variations in curriculum content, classroom discussions, and extracurricular opportunities that foster civic engagement.

### Reciprocal Effects of Efficacy and Participation

Traditional models of political participation, such as civic voluntarism (Verba et al., 1995) or the theory of planned behavior (Ajzen, 1991), posit that psychological dispositions predict political behavior. Accordingly, political efficacy has been viewed as a predictor of political participation. Early studies hypothesized that belief in the efficacy of voting would predict electoral participation (Campbell et al., 1954). To this day, many studies support this perspective, treating political efficacy as an antecedent of participation. Evidence from a U.S. sample of high school students suggested that both internal and external political efficacy predicted expected future political participation (Levy & Akiva, 2019). Moreover, the influence of internal efficacy was even stronger among students with high political interest. A German election survey with adults has demonstrated that internal political efficacy predicted conventional participation later, at three years and again at seven years (Reichert, 2016). A three-wave panel study conducted in Hong Kong with emerging adults found that sociopolitical control (i.e., perceived control, competence, and efficacy regarding one’s own socio-political context) predicted civic engagement (Chan & Mak, 2020). Similarly, analyses of two-wave panel data of adults in the U.S. have shown that internal political efficacy predicted political participation a few months later (Gil de Zúñiga et al., 2017). This study did not find similar results for external efficacy, however; while being inversely related to news consumption and discussion, external political efficacy had no effects on political participation.

While many studies support the idea that efficacy drives participation, alternative perspectives challenge this one-directional assumption. Drawing on Bem’s (1972) self-perception theory, some scholars argue that participation itself can shape efficacy. This theory proposes that individuals deduce their attitudes and emotions by observing their own behavior in context. When individuals lack strong internal reference points, they infer their attitudes based on their actions (Ikeda et al., 2008). Similarly, Bandura’s (1977) theory of self-efficacy highlights the role of experience in shaping beliefs about one’s own effectiveness. According to Bandura, self-efficacy beliefs develop through four key sources: mastery experiences, vicarious experiences, verbal persuasion, and physiological/emotional states. Among these, direct experiences (e.g., engaging in political activities) are particularly influential in shaping efficacy beliefs. However, the effect of participation on efficacy is likely conditional on the nature of the experience. Positive experiences, where individuals feel their actions have an impact, should reinforce a sense of political efficacy; meanwhile, negative experiences, such as frustration with bureaucratic obstacles or exclusion from decision-making, may weaken it (Beaumont, 2010). This distinction is particularly relevant when considering internal and external efficacy. Successful participation may indeed strengthen beliefs in one’s internal efficacy, but its impact on external efficacy likely depends on whether institutions and actors are perceived as responsive.

A longitudinal study among Belgian adolescents found that political participation had a stronger influence on political attitudes (i.e., political interest and political trust) over time than vice versa (Quintelier & Hooghe, 2012). The study further revealed that both individual and collective forms of participation contributed equally to these socialization effects, highlighting the potential of participation to shape political attitudes beyond initial predispositions. Regarding political efficacy, a Czech two-wave study with adolescents has shown that protest participation predicted higher internal political efficacy, as well as lower external political efficacy one and a half year later (Šerek et al., 2017). This suggests that, while experiences in non-institutionalized participation may enhance adolescents’ internal efficacy, it might simultaneously lead to perceptions of systemic unresponsiveness. Similarly, qualitative evidence from an interview study among Polish youth indicated a causal link between political activism, such as participation in climate protests, and increased political efficacy (Budziszewska & Głód, 2021). Conversely, a study with a nationally representative sample from Chile has found no relationship between social movement participation and group efficacy one year later (Smith et al., 2021).

From these findings, it becomes apparent that adolescence is a particularly relevant stage for examining the reciprocal relationship between political participation and political efficacy. This life phase is marked by increasing cognitive, social, and political awareness, alongside a growing capacity for independent action. Theory suggests that youth’s political development is a key task during this period (Wray-Lake, 2019). Adolescents are often engaging with political and social issues for the first time, which makes early experiences with participation potentially formative for their long-term political development. In recent years, non-institutionalized forms of political participation have gained relevance in the political life of young people. The inconsistencies observed in the research findings cited above underscore the necessity for further investigation. Grasping the dynamics of the relationship between political efficacy and non-institutionalized forms of political participation is therefore an important task in understanding adolescent political development. This is what this study proposes to do.

## Current Study

Political participation among youth is crucial for fostering active and informed citizens, yet non-institutionalized forms of participation, such as petitioning, demonstrating, and boycotting, are not fully understood in terms of their developmental impact. While previous research has focused on the predictors of political participation, the consequences and effects of such participation, especially during adolescence, remain underexplored. This study examines the relationship between non-institutionalized participation and political efficacy among high school students, controlling for gender, immigration background, and school track. Data from two measurement points (T1 = Grade 7; T2 = Grade 8) were used to test the hypothesis that internal and external political efficacy in Grade 7 would positively predict non-institutionalized participation in Grade 8 (Hypothesis 1). Furthermore, it is to be expected that non-institutionalized political participation in Grade 7 will lead to an increase in internal political efficacy (Hypothesis 2a) and a decrease in external political efficacy (Hypothesis 2b) by Grade 8. These hypotheses align with research suggesting a reciprocal relationship between efficacy and participation. Self-determined engagement with political topics may foster internal efficacy by reinforcing a sense of agency, while increased awareness of systemic limitations may lower external efficacy.

## Method

### Participants and Procedure

Data utilized in this study were obtained from the German EPKO panel study (Study on the development of political and societal competence in adolescence) initiated in the winter of 2021/22, beginning with data collection in Grade 7 and continuing in the winter of 2022/23 with Grade 8. The analyses focused on students who participated in the initial wave of data collection (*N* = 613) from classes involved in both waves (*N* = 25), resulting in a total sample of *N* = 444 students (wave 1: *M*_age_ = 12.94, *SD*_age_ = 0.61; 46.9% female). The study was conducted in North Rhine-Westphalia, Germany’s most populous state, encompassing rural, semi-urban, and urban settings across 10 schools and 25 classes.

Weighting by immigration background or gender was not necessary due to the data closely mirroring the distribution of students attending general education schools in North Rhine-Westphalia (IT.NRW, 2022). Participation required informed parental consent, resulting in response rates of 80 and 71% at waves I and II, respectively; non-participation was primarily due to lack of consent or student absence due to illness or quarantine measures.

### Measures

#### Non-institutionalized Forms of Participation

Non-institutionalized participation was measured as a latent construct, with three items forming a scale. Specifically, students were asked how often they had done the following activities: (1) “participation in a signature collection or online petition,” (2) “participation in a demonstration,” and (3) “rejection of certain products, for example, meat or products from certain countries, etc.” Participants rated each of the three activities on a scale from 1 to 4, with 1 meaning “never”, 2 “rarely”, 3 “sometimes”, and 4 “often”.

#### Internal and External Political Efficacy

Students’ internal and external political efficacy was assessed with the four-item scale Political Efficacy Kurzskala (PEKS, Beierlein et al., 2014) and two items from the German General Social Survey (ALLBUS) so that both types of efficacy were assessed with three items each. The introductory question for all items read as follows: “To what extent do the following statements apply to you?” Students were asked to rate each item on a four-point scale ranging from 1 (doesn’t apply at all) to 4 (applies very well). Example items were “I am good at understanding and assessing important political issues.” (internal) and “Politicians strive to keep in close touch with the people.” (external).

#### Control Variables

The variables used for control were immigration background, gender, and school track. For immigration background, students were asked which language they spoke at home (adapted from IGLU 2016, see Hußmann et al., 2020). For those students who reported speaking a language other than German at home, at least sometimes, an immigration background was assumed. In regards to students’ gender, male was coded as 0 and female as 1. As for school type, schools focused on vocational education were coded as 0, while schools preparing students for university entry were coded as 1.

### Data Analysis

All analyses were conducted using R version 4.3.2, primarily employing the *lavaan* package version 0.6–17 (Rosseel, 2012). Prior to the actual analyses, an attrition analysis was carried out. A logistic regression analysis was conducted to compare participants who participated in both waves of data collection to those who only took part in the first wave of data collection, based on all study variables at T1. Predictors with more than two indicators were modeled as latent variables in the analysis. Accordingly, measurement invariance analysis was conducted on internal and external political efficacy as well as non-institutionalized participation. Measurement invariance was assessed in four stages: configural invariance (same factor structure across time), metric invariance (equal factor loadings), scalar invariance (equal item intercepts), and strict invariance (equal residual variances). Models were estimated using robust maximum likelihood (MLR) in *lavaan* (Rosseel, 2012). Model comparisons were based on changes in comparative fit index (ΔCFI), root mean square error of approximation (ΔRMSEA), and standardized root mean square residual (ΔSRMR), with thresholds of ΔCFI ≤ |0.010 | , ΔRMSEA ≤ |0.015 | , and ΔSRMR ≤ |0.030| indicating invariance (Chen, 2007). To address the research question, two autoregressive cross-lagged panel models (i.e., one with and one without control variables) were run. All autoregressive paths were enabled, as were the cross-lagged paths for internal/external political efficacy and non-institutionalized participation. The models included correlations between the latent variables at T1 and residual correlations between the latent variables at T2. In addition, the models allowed for error correlations between indicators of the same latent variable across time points (i.e., parallel items). This means that the models allowed for correlations between the measurement errors of the same indicator at T1 and T2. This approach enabled the analysis of rank-order changes in political efficacy and participation, offering insight into individuals’ relative developments. The clustered structure of the data was accounted for by using class (*N* = 25) as the cluster variable, enabling the computation of cluster-robust standard errors. Given that students in the same class share a common educational environment, including the same teachers, classroom discussions, and instructional approaches, this clustering allowed for the introduction of dependencies in their responses. Ignoring this structure could have led to underestimated standard errors and inflated Type I error rates. With clustering at the class level accounted for, standard errors remain robust to within-class correlations, leading to more accurate statistical inferences. A one-way random-effect analysis of the intra-class correlation revealed that only 7, 1, and 6% of the variance in internal political efficacy, external political efficacy, and non-institutionalized political efficacy, respectively, were explained by the class structure in the sample. Together with the small sample size, this led to us to forgo multilevel analysis. Missing data were handled using the full information maximum likelihood (FIML) method. Additionally, sensitivity analyses were carried out, in which each non-institutionalized participation activity was treated as a separate outcome resulting in three additional models.

### Attrition Analysis and Measurement Invariance

Overall, 15.5% of the data was missing. Of the total sample (*N* = 444), 225 cases were complete, while 219 had missing data. The highest proportion of missing data occurred in the external political efficacy variable at T2, with a lack of 37.6%. This was primarily due to some students not participating in the second wave of data collection. To examine potential biases, a logistic regression analysis thus was conducted to compare T1 participants who did and did not take part in T2, based on all study variables measured at T1. The analysis revealed no significant differences, indicating that students who participated in T1 and T2 were comparable to those who only participated in T1. Additional logistic regression analyses were performed to assess whether missing responses to internal and external political efficacy, as well as non-institutionalized participation at T1, were systematically related to other study variables. No significant differences were found for internal and external political efficacy. Attending an academic-track school was significantly linked to responding to the non-institutionalized participation question. This is likely due to the placement of these items later in the questionnaire, which may have led students from non-academic-track schools to skip or not complete them. To examine whether the three latent constructs were measured equivalently across T1 and T2, measurement invariance was tested using a stepwise confirmatory factor analysis approach. The results demonstrated that strict measurement invariance was established for all three latent variables between Grade 7 and Grade 8. This allows for meaningful comparisons to be drawn between latent means and structural relationships across the two time points.

## Results

### Descriptives

Detailed information on the items and scales, including means, standard deviations, and correlations, is provided in Table 1. Internal political efficacy showed a strong correlation between Grade 7 and Grade 8 (*r* = 0.65, *p* < 0.001), while external political efficacy demonstrated a moderate correlation across grade levels (*r* = 0.38, *p* < 0.001). Non-institutionalized participation also exhibited moderate correlation between Grade 7 and Grade 8 (*r* = 0.49, *p* < 0.001). The correlations between the political efficacy scales and non-institutionalized participation revealed mixed results. Although all correlations were positive, only two reached statistical significance. Specifically, internal political efficacy in Grade 7 was significantly correlated with non-institutionalized participation in Grade 7 (*r* = 0.22, *p* = 0.013), and internal political efficacy in Grade 8 was significantly correlated with non-institutionalized participation in Grade 8 (*r* = 0.21, *p* = 0.025). These findings suggest that higher levels of internal political efficacy in both grades were associated with greater non-institutionalized participation within the same grade level. However, other correlations between political efficacy and participation did not reach statistical significance, indicating weaker or no associations. As expected, internal and external political efficacy were highly correlated in both grade levels (Grade 7: *r* = 0.69, *p* < 0.025; Grade 8: *r* = 0.54, *p* < 0.025). The observed difference in correlations between the two grade levels (Δ*r* = 0.15) may reflect the students developing a more nuanced and independent understanding of their own capabilities and the responsiveness of the political system as they mature. Yet, the difference between the two correlations was not statistically significant. The Cronbach’s alpha for internal political efficacy ranged from 0.80–0.81 across both grades, reflecting good reliability. External political efficacy exhibited reliability values between 0.69 and 0.71, while non-institutionalized participation demonstrated similar consistency, with α ranging from 0.70–0.71. These values suggest that the scales demonstrated adequate reliability across both waves of data collection. Additionally, the means for the constructs were relatively consistent across grades, with internal political efficacy and external political efficacy both having a mean of 2.3 across Grade 7 and Grade 8. Non-institutionalized participation had a mean of 1.6 in both grades, indicating rather low levels of participation in non-institutionalized activities.

**Table 1:** Means, standard deviations, and correlations

Variable	1	2	3	4	5	6	7	8	9
1. Internal political efficacy (Grade 8)	–								
2. Internal political efficacy (Grade 7)	0.65**	–							
3. External political efficacy (Grade 8)	0.54**	0.23**	–						
4. External political efficacy (Grade 7)	0.29**	0.69**	0.38**	–					
5. Non-institutionalized Participation (Grade 8)	0.21*	0.22	0.11	0.04	–				
6. Non-institutionalized Participation (Grade 7)	0.13	0.30**	0.01	0.11	0.49**	–			
7. Immigration background	−0.10	−0.10	0.07	−0.08	0.03	0.06	–		
8. School track (1 = academic track)	0.29**	0.25**	0.21**	0.16**	0.09	−0.07	−0.03	–	
9. Gender (1 = female)	−0.16**	−0.06	0.11	0.21**	0.11	−0.05	0.02	0.03	–
*M*	2.3	2.3	2.5	2.5	1.6	1.6	0.5	0.4	0.5
*SD*	0.7	0.7	0.6	0.7	0.7	0.7	0.5	0.5	0.5
Actual Range	1 – 4	1 – 4	1 – 4	1 – 4	1 – 4	1 – 4	0 – 1	0 – 1	0 – 1
*N*_items_	3	3	3	3	3	3	1	1	1
*N*_responses_	290	417	277	398	287	396	438	444	431
Cronbach’s α	0.81	0.80	0.69	0.71	0.70	0.71	–	–	–

*M* and *SD* represent mean and standard deviation, respectively

*p < 0.05. **p < 0.01

### Cross-lagged Effects Between Political Efficacy and Political Participation

The baseline model without control variables demonstrated a good model fit (CFI = 0.981, RMSEA = 0.025, SRMR = 0.051). All autoregressive paths were positive and statistically significant (see Table 2), indicating low to moderate rank-order stability over time (internal political efficacy: β = 0.63, *p* < 0.001; external political efficacy: β = 0.36, *p* < 0.001; non-institutionalized participation: β = 0.40, *p* = 0.004). This suggests that students who reported higher levels of internal political efficacy, external political efficacy, and non-institutionalized participation at T1 were more likely to maintain relatively higher levels of these constructs at T2 compared to their peers. However, the strength of stability varied across constructs. Internal political efficacy exhibited the highest rank-order stability (β = 0.63), indicating that students’ relative positions in this domain remained fairly consistent from Grade 7 to Grade 8. In contrast, external political efficacy showed the lowest stability (β = 0.36), implying that students’ relative standing in this construct was more prone to change over time. For the cross-lagged paths, no significant associations emerged. The strongest, though still non-significant, effect was found between internal political efficacy at T1 and participation at T2 (β = 0.17, *p* = 0.192). The explained variance in the latent constructs at T2 varied across variables. External political efficacy had the lowest explained variance at approximately 13%; participation accounted for 20%; and internal political efficacy demonstrated the highest explained variance, with 37% of its variance explained by its own baseline measure and other variables.

**Table 2:** Cross-lagged panel modeling results for political efficacy and non-institutionalized participation

Variable	Model 1 (Without control variables)	Model 2 (With control variables)		
*Intercept correlations*				
IPE ↔ NIP	0.30**	0.28**		
EPE ↔ NIP	0.12	0.10		
EPE ↔ IPE	0.68**	0.68**		
*Autoregressive Paths*				
IPE	0.63**	0.55**		
EPE	0.36**	0.33**		
NIP	0.40**	0.37**		
*Cross-lagged effects*				
IPE → NIP	0.17	0.29*		
EPE → NIP	−0.13	−0.26		
NIP → IPE	−0.05	−0.05		
NIP → EPE	−0.03	−0.03		
*Control variables*		W2 IPE	W2 EPE	W2 NIP
Immigration background		−0.07	0.08	0.02
School track		0.19**	0.16*	0.07
Gender		−0.14*	0.06	0.15
*N*	441	429		
CFI	0.984	0.932		
RMSEA	0.025	0.050		
SRMR	0.051	0.064		

*IPE* internal political efficacy, *EPE* external political efficacy, *NIP* non-institutionalized participation

*p < 0.05. **p < 0.01

The main model, which included control variables, demonstrated an acceptable model fit (CFI = 0.919, RMSEA = 0.046, SRMR = 0.064). As in the baseline model, all autoregressive paths remained positive and statistically significant (see Table 2), confirming moderate to high rank-order stability over time (internal political efficacy: β = 0.55, *p* < 0.001; external political efficacy: β = 0.33, *p* < 0.001; non-institutionalized participation: β = 0.37, *p* = 0.007). For the cross-lagged paths, internal political efficacy at T1 was significantly associated with non-institutionalized participation at T2 (β = 0.29, *p* = 0.034), suggesting that students with higher initial levels of internal political efficacy were more likely to report higher levels of non-institutionalized participation one year later. In contrast, external political efficacy at T1 was not significantly related to non-institutionalized participation at T2 (β = –0.26, *p* = 0.078). Similarly, participation at T1 was not associated with either internal political efficacy at T2 (β = –0.02, *p* = 0.783) or external political efficacy at T2 (β = –0.02, *p* = 0.755). Turning to the control variables, one finds that attending an academic-track school was significantly positively associated with internal political efficacy at T2 (β = 0.19, *p* = 0.006). Furthermore, attending an academic-track school was also significantly positively related to external political efficacy at T2 (β = 0.16, *p* = 0.024). Gender differences also emerged, in that being female was negatively associated with internal political efficacy (β = –0.14, *p* = 0.014).

### Sensitivity Analyses

The sensitivity analyses largely replicated the findings from the main model (see Table 3). All models demonstrated at least an acceptable model fit (petitioning: CFI = 0.918, RMSEA = 0.053, SRMR = 0.061; demonstrating: CFI = 0.923, RMSEA = 0.051, SRMR = 0.063; boycotting: CFI = 0.915, RMSEA = 0.055, SRMR = 0.074). In the petitioning model, the autoregressive paths for internal and external political efficacy were both positive and statistically significant. However, no significant autoregressive path was found for petitioning. For the cross-lagged paths, internal political efficacy at T1 was positively associated with petitioning at T2 (β = 0.28, *p* = 0.035). As for the control variables, attending the academic track was positively associated with both internal and external political efficacy, while being female was negatively associated with internal political efficacy. In the model measuring protest participation, the autoregressive paths for internal and external political efficacy were positive and statistically significant, but no significant autoregressive path was found for demonstrating. None of the cross-lagged paths reached statistical significance. Again, attending the academic track was positively associated with internal and external political efficacy, while being female was negatively associated with internal political efficacy. The boycott model revealed a similar pattern. Autoregressive paths for internal and external political efficacy were positive and statistically significant, but no significant autoregressive path was found for boycotting. For the cross-lagged paths, internal political efficacy at T1 was positively associated with boycotting at T2 (β = 0.28, *p* = 0.033). Attending an academic-track school was positively associated with internal political efficacy, external political efficacy, and boycotting at T2. Additionally, being female was negatively associated with internal political efficacy but positively associated with boycotting.

**Table 3:** Cross-lagged panel modeling results for political efficacy and petitioning, demonstrating, and boycotting

Variable	Model 3 (Petitioning)			Model 4 (Demonstrating)			Model 5 (Boycotting)		
*Intercept correlations*									
IPE ↔ NIP	0.17			0.23**			0.32**		
EPE ↔ NIP	0.09			0.06			0.23**		
EPE ↔ IPE	0.68**			0.68**			0.67**		
*Autoregressive Paths*									
IPE	0.56**			0.55**			0.53**		
EPE	0.32**			0.33**			0.32**		
NIP	−0.04			−0.01			−0.02		
*Cross-lagged effects*									
IPE → NIP	0.33*			0.30			0.33*		
EPE → NIP	−0.21			−0.25			−0.17		
NIP → IPE	−0.05			−0.03			0.06		
NIP → EPE	0.01			−0.03			0.01		
*Control Variables*	W2 IPE	W2 EPE	W2 NIP	W2 IPE	W2 EPE	W2 NIP	W2 IPE	W2 EPE	W2 NIP
Immigration background	0.08	−0.06	0.03	0.08	−0.07	−0.01	0.08	−0.06	0.03
School track	0.17*	0.18*	−0.04	0.16*	0.18**	0.05	0.16*	0.18**	0.19**
Gender	0.06	−0.14*	0.11	0.06	−0.14*	0.10	0.06	−0.15**	0.13*
*N*	429			429			429		
CFI	0.921			0.934			0.936		
RMSEA	0.052			0.047			0.049		
SRMR	0.058			0.056			0.058		

*IPE* internal political efficacy, *EPE* external political efficacy, *NIP* form of non-institutionalized participation

*p < 0.05. **p < 0.01

## Discussion

Understanding the relationship between political efficacy and political participation is crucial for grasping the dynamics of political development in youth. While prior research has often emphasized the role of political efficacy as a predictor of participation, less is known about whether participation itself fosters efficacy, particularly during adolescence. To address this gap, the present study has examined the bidirectional relationship between political efficacy and non-institutionalized forms of democratic participation, such as petitioning, demonstrating, and boycotting. The findings do not provide conclusive support for a reciprocal relationship. Instead, autoregressive effects suggest that internal political efficacy remains relatively stable over time. The only cross-lagged effect observed was that internal political efficacy in Grade 7 was positively associated with non-institutionalized participation in Grade 8, whereas no evidence was found for an effect in the opposite direction.

The autoregressive effects were moderate for internal political efficacy but low for external political efficacy and non-institutionalized participation. The greater stability of internal efficacy is consistent with prior research, suggesting that it represents a relatively enduring psychological trait shaped by early socialization and personal agency. Evidence from adult samples supports this interpretation, having shown strong autoregressive paths for psycho-political empowerment, a construct closely related to internal efficacy, indicating its persistence over time (Christens et al., 2011). Similarly, analyses of middle school students in the U.S. found moderate to strong autoregressive paths for political empowerment (Messman et al., 2022). In contrast, the lower stability of external political efficacy may result from its dependence on contextual factors, such as political events or institutional trust, which tend to fluctuate over time. Prior studies have indicated that external political efficacy is shaped by external influences, such as politicians’ actions and the design of government institutions, rather than being a stable, internalized trait (Wolak, 2018). Likewise, non-institutionalized participation appears to be somewhat situational, influenced by specific opportunities for engagement rather than reflecting a stable behavioral tendency.

Interestingly, sensitivity analyses revealed no significant autoregressive paths for any of the three individual types of non-institutionalized participation in this investigation (petitioning, demonstrating, and boycotting). However, the significant autoregressive path for the latent construct implies that, while the general propensity to participate remains relatively stable, the specific modes of engagement do fluctuate based on available opportunities and contextual influences. The absence of significant autoregressive paths for individual participation types underscores the importance of considering broader constructs when assessing political engagement trajectories in adolescence. The results suggest that, while some political dispositions consolidate during adolescence, others remain more fluid, highlighting this stage as a critical period for political development.

As for the cross-lagged paths, the results demonstrate that internal political efficacy in Grade 7 is associated with non-institutionalized participation in Grade 8. Given that non-institutionalized participation often requires individual initiative and effort, internal political efficacy can be expected to play a stronger role in motivating engagement than external efficacy (Park, 2019). Research suggests that fostering young people’s confidence in their capacity to create change, alongside providing tangible opportunities for engagement, can promote democratic behaviors and mitigate disengagement (Messman et al., 2022). However, no other cross-lagged effects proved statistically significant.

The absence of reciprocal effects is rather counterintuitive, as previous research has highlighted that self-determined, non-institutionalized forms of participation can provide individuals with a sense of autonomy and competence (Pickard, 2019, 2022). Several factors may explain why no effect from participation to efficacy was found. One possibility is that it remains unclear how invested students were when engaging in these non-institutionalized activities. Simply signing a petition without deeper engagement with the issue may not provide a strong enough experience to enhance political efficacy. Additionally, the nature of participation matters: Students who are externally motivated or minimally engaged may not experience the empowerment effects that come with meaningful civic involvement. Another potential explanation is that the one-year interval between measurements may be too long, since participatory experiences to fade over time. However, this explanation is not fully supported, as one would still expect those who were heavily invested in their engagement to report higher participation scores over time. Furthermore, concerns about insufficient variability in the constructs do not seem warranted, given the observed standard deviations and the presence of moderate autoregressive effects. Finally, theoretical assumptions regarding the mechanisms through which participation fosters efficacy may require further refinement. If these non-institutionalized forms of participation do not foster efficacy as expected, alternative forms of engagement that promote deeper involvement, such as voluntary service, may be more impactful. Future research should explore the specific conditions under which different types of participation influence political efficacy.

Although some hypothesized effects were not substantiated, other notable findings emerged that warrant further exploration. In particular, the analysis highlights the role of academic tracking and gender, which both appear to influence political efficacy through structural mechanisms. Students in the academic track demonstrated higher internal and external political efficacy, suggesting that educational pathways contribute to political empowerment (Hoskins & Janmaat, 2016). Alternatively, this disparity may reflect self-selection effects, where students from more politically engaged backgrounds enter educational tracks that reinforce these dispositions. This dual reinforcement process could have significant implications for social disparities in political engagement and warrants deeper examination. Additionally, gender differences emerged, with female students reporting higher external efficacy but lower internal efficacy. This pattern may reflect persistent societal stereotypes in which men are more likely to express self-confidence in public matters (Karv et al., 2022). Future studies should investigate how social background, educational opportunity structures, and gender norms intersect to shape political efficacy and participation.

Despite its strengths, this study did have some limitations. Although the use of two measurement points is an improvement over cross-sectional designs, more measurement points over shorter intervals would have better captured the developmental trajectory of political efficacy and participation (Arens & Watermann, 2017). The analyses focused on capturing rank-order changes rather than true within-person changes. While this approach provided insight into relative differences between individuals over time, it did not assess absolute growth within individuals. Accurately measuring individual development would have required more than two time points and the application of growth curve or change score models. Therefore, any observed increases should be understood as shifts in individuals’ relative positions rather than definitive personal growth. Additionally, the measures used in this study did not assess the intensity or specific nature of engagement, which may influence the strength of potential effects. Ideally, future studies should employ a more nuanced approach by differentiating between various types and intensities of political activities and by exploring which forms of engagement provide meaningful democratic learning opportunities. Moreover, the study was conducted in North Rhine-Westphalia, Germany, which may limit the generalizability of the findings to other cultural and political contexts. While North Rhine-Westphalia, as Germany’s most populous state, does provide a relevant case for studying youth political socialization, the findings should be interpreted with caution when applied to different educational systems or political cultures. Prior research has suggested that the relationship between political efficacy and participation is shaped by contextual factors, including the structure of political institutions, the extent of civic education, and the broader participatory culture in a given society (Oser et al., 2022). For instance, studies have indicated that adolescents in countries with high government transparency and strong civic education curricula report higher levels of political efficacy and engagement (Cicatiello et al., 2018; Feitosa, 2020), whereas in contexts with less developed civic contexts the efficacy-participation link may be weaker or function differently (Bolzendahl & Coffé, 2013). Similarly, educational systems vary in the extent to which they emphasize political engagement as part of the curriculum. Germany, for example, has a structured civic education framework, particularly in secondary schools, which may reinforce political efficacy more strongly than in countries with less formalized civic education. Additionally, political opportunity structures, such as the availability of youth councils, student representation, or grassroots activism, differ between nations and may moderate the reciprocal relationship between efficacy and participation.

Future research could benefit from expanding these findings by examining different forms of political participation, including both institutional and non-institutionalized activities, and exploring their varying levels of engagement intensity. Differentiating between types of participation, such as voting, volunteering, activism, and online engagement, could clarify how each contributes uniquely to political efficacy. Building on Bandura’s framework (1977), it is essential to recognize that individuals engaging in political participation may have diverse experiences, ranging from empowering to discouraging. The results of this study suggest that the nature of these experiences is pivotal; participation alone may not be sufficient to enhance efficacy unless it involves meaningful and self-directed engagement. Furthermore, the impact of these experiences likely varies across individuals, depending on personal predispositions and social influences. These considerations underscore the need for future research to examine which specific participatory experiences enhance political efficacy and how individual differences moderate these effects.

Moreover, intervention studies focusing on structured participatory activities, such as civics courses, youth councils, or political simulations, could provide further insights into causal pathways between participation and efficacy. Investigating whether targeted interventions foster long-term increases in political efficacy would contribute to a deeper understanding of how civic education can promote engagement. Finally, longitudinal research with shorter measurement intervals could capture the gradual changes in efficacy that longer intervals might miss, offering greater insight into how different participatory experiences contribute to democratic capacities. Schools could integrate participatory learning experiences into the curriculum, ensuring that students across all educational tracks have access to meaningful civic education and engagement opportunities.

## Conclusion

Political efficacy is widely recognized as a key determinant of political participation, yet its potential to be shaped by participatory experiences during adolescence remains insufficiently understood. While prior research has established that efficacy predicts engagement, less is known about whether participation, particularly in non-institutionalized forms, reciprocally enhances efficacy. Addressing this gap, this study examined the longitudinal relationship between political efficacy and participation among students, focusing on the transition from Grade 7 to Grade 8. The findings indicate that while internal political efficacy positively predicts subsequent petitioning, there is no evidence for a reciprocal effect of participation on efficacy. This suggests that, within this developmental period, political efficacy is a relatively stable trait that guides engagement rather than being significantly shaped by it. These results contribute to our understanding of political socialization in early adolescence by highlighting the limited short-term impact of participation on political efficacy.
